# N-acetylcysteine dose-dependently improves the analgesic effect of acetaminophen on the rat hot plate test

**DOI:** 10.1186/s40360-020-00469-4

**Published:** 2021-01-07

**Authors:** Samaneh Nakhaee, Mohammad Dastjerdi, Hesam Roumi, Omid Mehrpour, Khadijeh Farrokhfall

**Affiliations:** 1 Medical Toxicology and Drug Abuse Research Center (MTDRC), Birjand University of Medical Sciences (BUMS), Birjand, Iran; 2 Cardiovascular Research Center, Birjand University of Medical Sciences, Birjand, Iran; 3 Mel and Enid Zuckerman, College of Public Health, University of Arizona, Tucson, Arizona USA

**Keywords:** Acetaminophen, N-acetyl cysteine, Analgesic effect, Hot plate, Co-administration, Pain, Rat

## Abstract

**Background:**

Acetaminophen (APAP) induced hepatotoxicity is a clinically important problem. Up to now, interventive therapy with n-acetylcysteine (NAC) has been considered as a gold-standard treatment for APAP overdose. However, no study has focused on the efficacy of these drugs’ concurrent administration on probable enhancing therapeutic outcomes. Thus, this study was aimed to investigate the analgesic effect of co-administration of NAC and acetaminophen in male rats. The NAC-APAP drug formulation may demonstrate the stranger antinociceptive effect.

**Methods:**

Forty-eight male Sprague-Dawley rats (12–14 weeks) randomly divided into six equal groups; control, APAP (received 300 mg/kg APAP), NAC (received 600 mg/kg NAC) and APAP+ NAC groups that received simultaneously 300 mg/kg APAP with 200–600 mg/kg NAC (AN200, AN400, AN600). All administrations were done orally for once. The antinociceptive effect was recorded by measurement of latency period on a hot plate in 30, 60, and 90 min after administrations.

**Results:**

The results showed that NAC’s concurrent administration with APAP, dose-dependently increased APAP analgesic effects (*p*< 0.0001). Moreover, NAC treatment exhibited an antinociceptive effect in 60 and 90 min, per se. The treatments had no adverse effect on liver enzymes and oxidative stress.

**Conclusion:**

Co-administration of NAC with APAP can improve the antinociceptive effect of APAP. It is suggested that this compound can enhance analgesic effects of APAP and eventually lead to a reduction in acetaminophen dose. Further studies are needed to evaluate the molecular mechanism of this hyper analgesic effect.

## Background

Paracetamol (the international name of the medicine in Europe) and Acetaminophen (N-acetyl-p-aminophenol*, APAP,* the international name of this medicine in the US) are both the official names of a chemical compound [1]. Morse developed this drug for the first time in 1878, and Von Mering used it clinically in 1887 [2]. Acetaminophen is an antipyretic and analgesic drug that can be bought and used without prescription worldwide. Its antipyretic and analgesic properties are due to inhibition of prostaglandin production, similar to that of NSAIDs, but unlike them, APAP does not influence gastric mucosa; therefore, it does not cause side effects within the recommended dosage. The dosage of APAP greater than the maximum recommended dosage can increase the risk of liver damage. Although the rate of unwanted side effects of this drug has been reported to be low, APAP poisoning is considered the cause of at least 42% of severe liver failures observed in the United States’ intensive care units [3]. N-acetylcysteine, which is usually known in the form of acetylcysteine, is considered a non-toxic drug. Mathew and Prescott first recommended NAC as an antidote for hepatotoxicity resulting from APAP in 1974 [4]. This substance acts as an antioxidant, mucolytic, and anti-inflammatory factor—otherwise, oxidative stress encourages central pain transmission [5]. Studies have shown that antioxidant substances can reduce pain transmission in human and rodent pain models [6–9]. Previously, Mehrpour et al. in 2011 suggested that the combination of APAP with NAC can be suitable in making drug formulation and may cause a significant decrease in its poisoning rate [10]. This idea recently has been received attention [11]. The Owumi study has shown that APAP’s combination with NAC prevented liver injury due to APAP overdose [12]. However, no study has focused on the analgesic efficacy of APAP in combination with NAC. Therefore, the present study was carried out to investigate the analgesic effect of taking oral APAP and NAC simultaneously.

## Methods

### Experimental groups and drugs administration

This study was conducted on forty-eight 12–14 weeks-old male Sprague-Dawley rats (considering the pilot experiment) with a weight range of 200 to 250 g, obtained from Research Centre of Experimental Medicine, Birjand University of Medical Sciences.

All animals included in the study were healthy and of the same species and gender, with normal behaviors. Rats that had been previously used in other experiments were not included. Exclusion criteria were considered as death during experiments and animals with aberrant behavior (*n*=0). The rats were kept in a 12 h light/dark cycle room with a temperature of 22 ± 2 ͦ C, in the animal house of Birjand University of Medical Sciences (BUMS). The animals had free access to food and water. The study was performed in accordance with the guidelines of experimental animals approved by the ethics committee of BUMS (No; ir. bums: rec.1397.197). The rats were kept for 2 weeks in the laboratory for adaptation. After 12–16 h of fasting, the basal hot plate assay was performed, and the rats were then randomly divided into six equal groups. The calculation of the sample size is the basis of the mathematical formula for the “group comparison, repeated measurements” design for animal studies [13]. Similar to the other study, there were eight rats in each group [14, 15]. Different treatments were given to various groups as follows:

Group 1: treated with normal saline (control).

Group 2: treated with acetaminophen 300 mg/kg (APAP).

Group 3: treated with n-acetylcysteine 600 mg/kg (NAC).

Group 4: treated with acetaminophen 300 mg/kg and n-acetylcysteine 200 mg/kg (AN 200).

Group 5: treated with acetaminophen 300 mg/kg and n-acetylcysteine 400 mg/kg (AN 400).

Group 6: treated with acetaminophen 300 mg/kg and n-acetylcysteine 600 mg/kg (AN600).

**Fig. 1:**
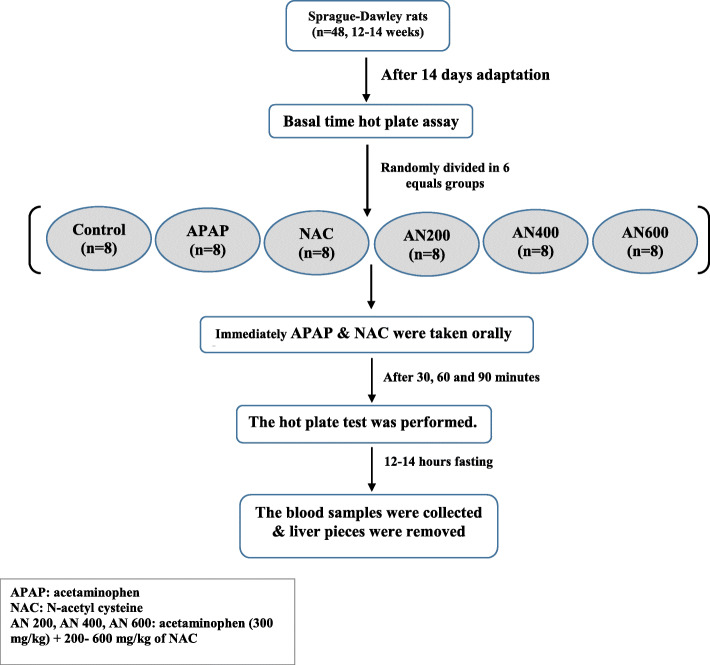
process flow diagram for the planning and performance of animal experiments

Feeding was done using steel gavage catheters and insulin syringes 30 min. Before algesimetric hot plate assay (time 30). The maximum volume of gavage did not exceed 10 ml/kg (about 2 ml). One day after the behavioral experiment; the animals were profoundly anesthetized by ketamine/ Xzylazin (80/10 mg/kg), intraperitoneally before euthanization and blood samples were collected from the heart. An equal piece of the liver was also sampled, washed with cold normal saline, and stored at − 80 °C until use. The flow chart was presented in Fig. 1.

### Biochemical evaluation

The plasma samples were assayed to measure liver enzyme concentrations (ALT, AST) and glucose level using commercial kits. The liver samples were homogenized in 10 volumes of cold PBS (phosphate buffer saline, pH 7.4) by a homogenizer (Miccra D-1, Germany). Next, the homogeneous tissue was centrifuged at 15000 rpm for 20 min [16]. The levels of ALT, AST, and MDA were then measured in the extract tissues.

### Thiobarbituric acid reactive substance (TBARS) assay

The samples of homogeneous tissue were provided, as explained in the previous section. The analysis was done based on the pre-explained method [17] on the basis of thiubarbituric acid’s reaction in acidic pH at 90–100 °; a spectrophotometer measured C. Absorption of the pink product at 535 nm. Malondialdehyde (MDA) is considered to be the equivalent of TBARS.

### Algesimetric assay

The hot plate test was carried out using an analgesiometer (Borjsanatazma. Tehran, Iran). This apparatus consisted of an electrically heated surface at a constant temperature of 55 ±0.5 ͦ C. The latencies for paw licking (or jumping) of the rats were assessed with the hot-plate test at 30 (time 30), 60 (time 60), and 90 (time 90) minutes after drug administration by the same investigator blinded to group allocation. The reaction time was considered the interval between the moment the animal was placed on the plate and the time it started licking its claws or raising its hands [18]. The cut of time point was considered 24 s [19], and the animals which showed no reaction at this time point were excluded from the study.

### Drugs

Acetaminophen solution vials were obtained from Alborz Darou Company (Qazvin Province, Alborz Industrial City, Iran). N-acetyl cysteine vials were obtained from Exir Company (Tehran Province, Tehran, Iran). The ketamine and Xzylazin vials were purchased from Rotexmedica, Germany, and Alfasan, Netherland, respectively. PBS tablets and thiobarbitoric acid were obtained from Sigma Laboratories. ALT, AST, and the glucose were measured by commercial kits constructed by Pars Azmun Company (Tehran, Iran).

### Statistical analysis

The descriptive statistics for the studied groups have been presented in the form of a mean ± standard deviation. Investigating the normal distribution of data was done through the D’Agostino & Pearson omnibus normality test. In order to compare the reaction time to heat stimulus, the one-way ANOVA test was used, followed by the Bonferroni post hoc test. Repeated measures of ANOVA were performed for within-group comparisons of various time points. The effect size in results means the rate of volubility of data in the application. Small effect; ≤ 0.2, moderate effect; ≤ 0.5, and large effect; ≥ 0.8 [20]. It is noteworthy that using the GraphPad Prism software version 7 (GraphPad, California USA) and considering the confidence coefficient of 95%, the abovementioned statistical analyses were carried out. Effect size in calculating sample size was obtained from similar studies of 6–10 rats per group [15, 21].

## Results

**Fig. 2:**
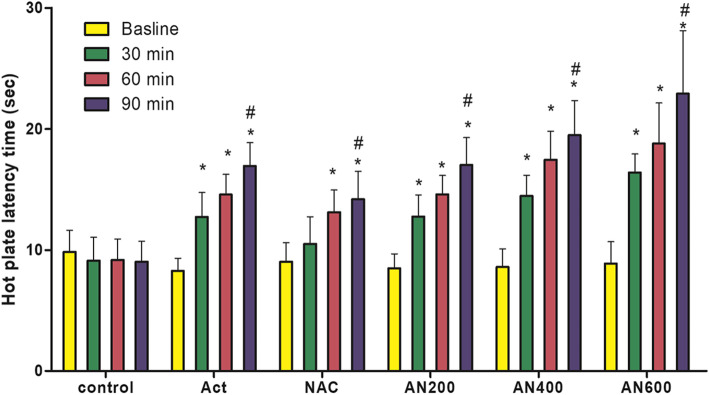
changes of analgesia with duration (baseline (0 min), 30–90 min after intervention) in control saline group (control) acetaminophen (APAP), N-acetylcysteine (NAC) and APAP (300 mg/kg) combination with the various doses of NAC 200–600 mg/kg of (AN 200, AN 400, AN 600). In each group, data in baseline and consecutive three times were analyzed by repeated measure. The data have been reported by mean ± standard error. *; significant compare with the baseline, #; significant 90 min compare with the 30 min

Forty-eight male Spague-Dowley rats (mean of body weight ± SEM; 237.3± 4014, 235± 2.11, 236.9± 3.12, 228.8± 5.4, 228.1± 4.02 and 238.1± 3.72 g in control, APAP, NAC, AN200, AN400, and AN600 respectively) were randomly allocated into six groups of 8 rats each. All acquired data were included in the statistical analysis. In the present research, baseline values for the control, Act, NAC, AN 200, AN 400, AN 600, and control groups were not significantly different. The average time of response to the heat stimulator (the hot plate) within 30, 60, and 90 min after receiving the interventions was calculated and compared in the experimental groups. The paw latency period in the various groups was analyzed and presented in Table 1, and the dose-dependent analgesic effect of NAC associated with 300 mg/kg APAP (200-600 mg/kg) was presented in Fig. 2.

**Table 1:** Paw latency period on hot plate test in the experimental groups (*n*= 8 in each group)

	Control	APAP	NAC	AN 200	AN 400	AN 600	Effect size	CI (95%)
Baseline	9.83 ±1.8	8.27 ±1.1	9.2 ±1.6	8.48 ±1.18	8.6±1.5	8.88±1.89	0.01	0.08–0.15
30 min	9.13±1.92	12.37±2.01^a^	10.50±2.24^b^	12.76±1.77^ac^	14.46±1.69^ac^	16.40±1.53^abcd^	0.65	0.55–0.78
60 min	9.18±1.71	14.58±1.68^a^	13.11±1.83^a^	14.59±1.57^a^	17.45±2.36^abcd^	18.79±3.35^abcd^	0.70	0.67–0.75
90 min	9.04±1.67	16.95±1.91^a^	14.20±2.29^a^	17.2±2.26^a^	19.49±2.85^abc^	22.91±5.19^abcd^	0.71	0.67–0.79

The analgesic effect of acetaminophen (APAP), N-acetylcysteine (NAC) and simultaneous feeding of acetaminophen (300 mg/kg) with the dosage of 200–600 mg/kg of NAC and comparing that with the control group in rats. There were 8 Sprague-Dawley rats in each group. Delay periods in the all groups were analyzed through the one-way ANOVA test followed by the Bonferroni post hoc test. The data have been reported by mean± standard deviation

^a^significant difference with the control group

^b^significant difference in comparison with APAP

^c^significant difference in comparison with NAC

^d^significant difference in comparison with AN 200

### Antinociceptive activity

The results revealed that APAP at the dose of 300 mg/kg could produce a significant antinociceptive effect at all time periods (30–90 min) compared to the control group. Moreover, the NAC 600 mg/kg only imposed analgesic effect 60 and 90 min. After taken. Values were not significantly different from those obtained in the APAP group, but had a significant difference to those of the control group. The different doses of NAC (200–600 mg/kg) and APAP (300 mg/kg) significantly increased the latency period compared to the control and NAC groups. However, APAP combined with 200 mg/kg NAC did not exhibit significant antinociceptive activity 30 min after administration. On the other hand, NAC (600 mg/kg) with APAP (300 mg/kg) improved the analgesic effect, and values were significantly higher than the APAP group (Table 1) 60 and 90 min. After consumption.

NAC, in a dose-dependent manner, could improve the analgesic effect of APAP. While the AN 600 group’s antinociceptive potential was significantly higher than the AN 200 group in all the studied time points, the AN 400 group had significantly higher antinociceptive effects than the AN 200 group at 60 and 90 min time points, but not after 30 min of administration.

There was no statistical difference between AN 600 and AN 400 in all the studied time points (Table 1).

### Time-dependent of analgesic effect

The pattern of antinociceptive efficacy of all investigations increased time-dependently. However, the pattern for the NAC group began after 30 min. In addition, the combination of APAP and NAC could significantly increase analgesia more efficiently after 90 min compared to after 30 min. Regarding the time period of 60 min compared with that of 30 min, a significant difference was observed only in the NAC group (Fig. 2).

### The effect of various treatment on biochemical assays

Furthermore, Table 2 demonstrates the effect of NAC and acetaminophen on serum values of glucose, AST, ALT activity in serum and liver, and MDA in the homogeneous liver tissue. No significant difference was found between the experimental groups within the parameters as mentioned above.

**Table 2:** Biochemical parameters of serum and liver in the experimental groups

Groups	Groups (Mean ± SD)					
	FPG (mg/dl)	Plasma ALT (IU/L)	Plasma AST (IU/L)	Liver ALT (IU/L)	Liver AST (IU/L)	Liver MDA (μmol/L)
**Control**	113.9 ±16.98	44.26 ±10.33	153.8 ± 42.11	173,700±45,540	91,180 ± 15,490	8.72 ± 1.46
**APAP**	95.10 ± 28.76	59.63 ± 15.56	158.8 ± 28.06	167,200± 50,750	92,420 ± 12,430	8.45 ± 2.39
**NAC**	104.0 ± 29.85	50.62 ± 3.765	137.7 ± 25.91	146,300± 30,530	87,420 ± 16,930	9.44 ± 0.99
**AN 200**	84.87 ± 36.98	57.30 ± 6.715	163.4 ± 11.25	181,200± 37,140	110,600 ± 16,930	6.91 ± 1.06
**AN 400**	101.2 ± 31.83	54.92 ± 11.18	171.4 ± 22.01	174,000± 35,010	97,150 ± 13,560	8.30 ± 3.13
**AN 600**	102.4 ± 38.89	60.37 ± 7.842	153.1 ± 25.58	162,900± 33,460	97,950 ± 20,470	9.81 ± 3.17

The serum and liver level of liver enzyme activity and lipid peroxidation in control saline group (control) acetaminophen (APAP), N-acetylcysteine (NAC) and acetaminophen (300 mg/kg) combination with the various dosage of 200–600 mg/kg of NAC (AN 200, AN 400, AN 600) in rats. There were 8 Sprague-Dawley rats in each group. The data have been mentioned by mean± standard deviation. Each data in the all groups were analyzed through the one-way ANOVA. There were no significant differences

## Discussion

In this study, the APAP’s therapeutic dose associated with various healthy doses of NAC in rat acute/thermal/supraspinal pain model using the hot plate test was investigated. The results showed that NAC accompanying APAP consumption improved APAP’s antinociceptive effect without any adverse effect on blood glucose, oxidative stress, and liver enzyme activity.

### Both NAC and APAP have analgesic effects in thermal pain induced by hot plate

Previous studies confirmed that the APAP dose-dependently has an antinociceptive effect in rats [14, 22, 23]. Previous studies have shown that a dose of 300 mg/kg of APAP is effective in controlling hot plate pain in rats [15, 23]. A hot plate test is used to check central pathways of the pain. Considering that APAP’s analgesic effect is applied by central effects with little or almost no effect on the periphery, a hot plate test is frequently used for this purpose [24]. The non-nociceptive factors, such as weight, also influence paw withdrawal responses in the hot plate test. In detail, weight has an inverse correlation with the latency period in baseline time; the heavy animals show a shorter baseline latency period [25]. Thus, animals with similar (234±7.33) body weights were used in our study. In this study, the APAP imposed a time-dependent analgesic effect, which is in agreement with the previous studies as well as the APAP pharmacokinetics [23, 26, 27].

The present study’s results clearly showed that NAC at the dose of 600 mg/kg exhibited analgesic activities mainly at 60 min and 90 min after administration. These NAC doses (200–600) are safe, and there is evidence showing that the LD50 of NAC in rats is 1205 mg/kg [28]. In line with our findings, NAC also imposed antinociceptive effects in the other studies [6–8, 29–31]; NAC can attenuate neuropathic and inflammatory pain in animal models measuring by hot plate and formalin tests [6, 8, 29, 31], and chronic apical lesion in humans evaluating by the VAS (visual analog scale) pain score [32]. Also, single-dose and chronic administration of NAC increases delay time in hot plate test [9]. Moreover, it has been reported that NAC had an analgesic effect only after 30 min intraperitoneal injection with the same doses (200–600 mg/kg) on plantar test in rat [6]. However, our study showed that NAC’s analgesia appeared 1 h after consumption and continued for a longer period of time in the hot plate test. Administration route (orally instead of IP injection) could influence its efficacy [33]. Many pain relief mechanisms have been proposed for NAC, partly due to antioxidant effect [8], NO reduction on spinal level [7], and other mechanisms such as metabotropic glutamate receptor activation in rodents [9] and humans [34].

### N-acetylcysteine combined with acetaminophen consumption improved the antinociceptive potency of acetaminophen due to its central effects

Free radicals contribute to maintaining thermal hyperalgesia [35] facilitation of excitatory synapses transmission in spinal dorsal horns, partly by TRPV1 and TRPA1 channels [5]. We cannot find any study about the analgesic effect of concurrent use of NAC-APAP. In this study, the co-administration of APAP with NAC increased APAP analgesic potency. In this investigation, the underlying mechanisms involving the analgesic effects were not assessed. However, the NO or TRP pathways appear to be involved because these common pathways are manipulated by APAP and oxidative stress/NAC in the central level [5, 8, 31, 36, 37]. NAC is an antidote of APAP toxicity, high consumption of APAP for pain control, and increasing liver insufficiency prevalence; the co-administration of these compounds in one formulation will be much more valuable.

### Combination of NAC with APAP has no unwanted effect on liver function test, plasma glucose, or oxidative stress

Acetaminophen, NAC, and their combination did not influence blood glucose and liver transaminases and did not induce oxidative stress. Oxidative stress alters carbohydrate metabolism [38, 39]. The effect of NAC consumption on glucose metabolism is maintained controversial from hypoglycemic to no significant effect [5, 8, 31, 36, 37]. In this study, NAC itself and combined with acetaminophen did not cause any plasma glucose disturbance in fasted animals.

The liver enzyme’s activities in serum and liver were assayed. It is worth mentioning that ALT and AST’s serum level are the most common clinical indicators for examining hepatotoxicity [39]. Surprisingly, APAP did not increase ALT and AST levels in serum and liver homogenate. This study’s results contrasted with another study, which found that APAP with a 300 mg/kg dose could increase the enzyme levels [23]. It has been shown that Sprague-Dawley rats are resistant to liver impairment due to APAP consumption. Even higher dose APAP to 1 g/kg could not induce liver complications [40]. It is worth noting that APAP consumption could not create oxidative stress either.

This study has some limitations that should be taken into consideration when interpreting the data. First, based on the animal study design, the findings can only be considered indicative rather than representative of humans’ clinical situations. Second, we did not assess APAP’s effectiveness in different animal pain models (inflammatory/ neuropathic pain). Further experimental research is required to reveal the possible molecular mechanisms by which NAC improved the APAP analgesic effect.

## Conclusion

The results of the present study show that NAC has an analgesic effect possibility, with a central effect. Also, co-administration NAC with APAP improved analgesia, which is valuable in the clinical setting and pharmacy. Further studies are needed to confirm these results and to elucidate the involved pathway.

## Data Availability

The datasets used and/or analyzed during the current study available from the corresponding author on reasonable request.

## Abbreviations

APAP: Acetaminophen
ALT: Alanine Transaminase
AST: Aspartate Transaminase
MDA: Malondialdehyde
NAC: N-acetylcysteine
NO: Nitric Oxide
PBS: Phosphate Buffer Saline
TBARS: Thiobarbituric acid Reactive Substance
